# Sensing Enhancement on Social Networks: The Role of Network Topology

**DOI:** 10.3390/e24050738

**Published:** 2022-05-22

**Authors:** Markus Brede, Guillermo Romero-Moreno

**Affiliations:** 1 School of Electronics and Computer Science, University of Southampton, Southampton SO17 1BJ, UK; 2 School of Informatics, University of Edinburgh, Edinburgh EH8 3FF, UK; guillermo.romeromoreno@ec.ac.uk

**Keywords:** complex networks, opinion dynamics, sensing enhancement, collective decision making, social learning, collective intelligence

## Abstract

Sensing and processing information from dynamically changing environments is essential for the survival of animal collectives and the functioning of human society. In this context, previous work has shown that communication between networked agents with some preference towards adopting the majority opinion can enhance the quality of error-prone individual sensing from dynamic environments. In this paper, we compare the potential of different types of complex networks for such sensing enhancement. Numerical simulations on complex networks are complemented by a mean-field approach for limited connectivity that captures essential trends in dependencies. Our results show that, whilst bestowing advantages on a small group of agents, degree heterogeneity tends to impede overall sensing enhancement. In contrast, clustering and spatial structure play a more nuanced role depending on overall connectivity. We find that ring graphs exhibit superior enhancement for large connectivity and that random graphs outperform for small connectivity. Further exploring the role of clustering and path lengths in small-world models, we find that sensing enhancement tends to be boosted in the small-world regime.

## 1. Introduction

Aggregation into groups is a trait that is common in the animal world and can bestow evolutionary advantages. In swarms, flocks, herds, and packs, animals coordinate their behaviour and achieve an enhanced performance in varied survival activities such as defence [1,2], foraging [1,3,4], or migration [4,5]. However, successful group behaviours require specific coordination mechanisms, such as collective decision making [6], flocking [7], or task allocation [8]. Interestingly, coordination in most cases is not orchestrated by central planners with specific goals, but rather emerges from simple behavioural rules of individuals that can be easily evolved and lead to behaviour typical of complex systems [9].

Sensing the environment is one of the most important tasks in animal survival [10,11], as it is paramount for detecting risks [1] and finding resources [3,4] or shelter such as good locations for nests [12]. However, individuals are often limited in their cognitive capabilities, which may result in incorrect or incomplete information. By sharing information about the perceived quality of an attribute, groups can improve the accuracy of their sensing in a way akin to the distributed processing of information [7]. Such group sensing is also typical for humans, the difference being in the complexity and flow of information compared to most animal species. This is particularly true in contexts where information is hard to obtain or very complex, or where combining second-hand information from peers may be an efficient strategy to access environmental information [13]. A well-known mechanism that shows this type of group sensing in humans is the so-called *wisdom of the crowds*, where the mean values of individuals’ perceptions of a quality variable tend to be surprisingly close to the real value [14].

Researchers from the swarm robotics field have taken inspiration from group sensing in animal groups to incorporate similar mechanisms into swarms of robots [15,16,17]. For instance, some works have reproduced nest searching mechanisms of bees or foraging mechanisms of ants in swarms of robots, and have mathematically studied the positive feedback dynamics that form in so-called *best-of-n* problems [18,19]. They study the effect that different communication rules have on collective decision outcomes, leading to accurate yet slow arrival to a collective consensus using voter-model-like rules [20] or a faster convergence but error-prone collective decisions with majority dynamics, e.g., in updating rules [19]. Overall, there is strong interest in optimal designs of sensing enhancement in applications of “swarm engineering” [15].

Other recent works in this field have focused on the swarm sensing of a single binary state in dynamic environments, exploring under which conditions communication rules enable a collective to follow changes in the environment [21,22,23,24]. In these models, individuals can either individually determine the state of the environment on their own or make up their minds by aggregating group opinions. In this context, the work of [21,22] has shown that adopting a tuned trade-off between both ways of information gathering is important, as the best performance is achieved near the bifurcation point of a bi-stable system [21,22,25].

Most of the above studies either have assumed well-mixed populations where any two individuals are expected to meet with equal likelihood [18,19,22] or have modelled spatially embedded communication channels in the context of robot simulations [23]. However, unlike in animal environments, social structures in human systems tend to have well-defined structures that are not necessarily spatially correlated and that may affect collective sensing capabilities [26]. The issue of how social network structure can influence decision making abilities of a population gains additional relevance through our increasing use of social media to source news and political information [27] and the “news-finds-me” perception, i.e., the tendency of social media users to rely on being informed about current events through peer communication rather than by actively following the news [28].

Studying the effects of a groups’ social network topology on its sensing abilities is the main aim of this paper, which is based on a paper presented at the Conference on Complex Networks and their Applications in 2021 [29]. Based on the conference paper, we developed a simplified version of the modelling framework proposed by [22] and use analytical and numerical methods to analyse the sensing abilities of collectives in dynamic environments. We then extended the analysis to different types of complex social networks, exploring the effects of degree heterogeneity and spatial structure on collective sensing. Additional to findings already presented at the conference, the present paper has been reworked in terms of the positioning of results relative to the literature and backs up findings from the conference paper with some scaling analysis on system size. Furthermore, we introduce extensions in two other important ways. First, in Section 4.2, we add to previous analyses by investigating the role of small-world networks and find that optimal sensing enhancement is typically achieved for small densities of shortcuts in the small-world regime. Second, results are generalised to a setting of environments with n>2 possible states. The effects of a larger number of states are discussed in Section 4.3.

## 2. Model Description

Let us consider a group of agents that aim to perceive a specific discrete feature $S_{t}\in \left\{1,2,\ldots ,n\right\}$ from a dynamic environment which can be in one of *n* possible states. At each time step, each agent holds a belief $s_{i}\left(t\right)\in \left\{1,\ldots ,n\right\}$ about the current state of the environment. As a basic mechanism, agents can directly measure the environment to update their beliefs. Individual updating is done via imperfect sensors with accuracy 1/n<q<1, meaning that, at each measurement, an agent will retrieve the correct state of the environment S(t) with probability *q* and an incorrect state with probability 1−q. When sensing incorrectly, we assume that an agent’s sensors retrieve each of the incorrect states with equal likelihood, i.e., $P(s_{i}\left(t+1\right)\neq S\left(t+1\right))=\left(1-q\right)/\left(n-1\right)$. Note that one could easily also consider other models of incorrect sensors that may favour a particular incorrect signal, but for the purposes of this paper, we will restrict analysis to the case of equal likelihood of error states. As an alternative to individual sensing, agents can also pool and aggregate the beliefs of their neighbours to update their own beliefs, thus incorporating knowledge accumulated by their peers. The neighbourhood of an agent is determined by the communication structure of the population, which is represented by an adjacency matrix *A* whose values $a_{ij}\in \left\{0,1\right\}$ encode whether a communication channel exists between agents *i* and *j*—e.g., a range of vision in a school of fish, or following patterns in social media. We assume the aggregation of neighbours’ beliefs to follow complex-contagion-like voter dynamics [30], i.e., adoption of the belief $s_{i}=b$ by agent *i* due to peer communication happens with a probability equal to

(1) $$P\left(s_{i}\left(t+1\right)=b\right)=\frac{{\left(\frac{1}{k_{i}}\sum_{j}a_{ij}\delta_{s_{j}\left(t\right),b}\right)}^{\alpha }}{{\left(\sum_{b=1}^{n}\frac{1}{k_{i}}\sum_{j}a_{ij}\delta_{s_{j}\left(t\right),b}\right)}^{\alpha }},$$

where $k_{i}=\sum_{j}a_{ij}$ represents the in-degree of node *i*, α≥1 is an exponent measuring the strength of complex contagion as in [30], and δ is the Kronecker delta, with $\delta_{i,j}=1$ if i=j and $\delta_{i,j}=0$ otherwise. A value of α=1 retrieves the linear voter model dynamics, where the probability of adopting a state is proportional to the number of neighbours holding that state. As the value of α increases, preferences towards the majority emerge, retrieving the deterministic *majority rule* when α→∞.

We consider the state-updating dynamics to unfold in discrete time steps, at each of which every agent of the population synchronously decides between sensing the environment (with probability *p*) or aggregating information from neighbours (with probability 1−p). The parameter *p* thus describes the agent’s trade-off between individual sensing and group sensing. Additionally, at each time step, the environment may swap its state, S(t+1), with probability *u*, where *u* characterizes the speed of environmental change. Below, settings of u=0 correspond to a static environment and for u=1 the environment changes state at every updating step. Environmental change is thus described by

(2) $$S\left(t+1\right)=\left\{\begin{array}{ccc} S(t) & \mathrm{with}\;\mathrm{probability} & 1-u\\ \mathrm{any}\;\mathrm{element}\;\mathrm{of}\;\bar{S}\left(t\right) & \mathrm{with}\;\mathrm{probability} & \left(1-u)/(n-1\right) \end{array}\right.,$$

where $\bar{S}\left(t\right)=\left\{1,\ldots ,n\right\}-S\left(t\right)$. Here we have again assumed that all states in the environment occur with the same likelihood.

According to the dynamics described above, the expected probability of agent *i* to hold a belief $s_{i}\left(t+1\right)=S\left(t+1\right)$ about the environment at time t+1 is determined by

(3) $$P\left(s_{i}\left(t+1\right)=S\left(t+1\right)\right)=pq+\left(1-p\right)\frac{{\left(\sum_{j}a_{ij}\delta_{s_{j}\left(t\right),S_{i}\left(t+1\right)}\right)}^{\alpha }}{\sum_{b=1}^{n}{\left(\sum_{j}a_{ij}\delta_{s_{j}\left(t\right),b}\right)}^{\alpha }}\;,$$

whereas the probability to hold an incorrect belief $b\in \bar{S}\left(t+1\right)$ is given by

(4) $$P\left(s_{i}\left(t+1\right)=b\right)=p\left(1-q\right)/\left(n-1\right)+\left(1-p\right)\frac{{\left(\sum_{j}a_{ij}\delta_{s_{j}\left(t\right),b}\right)}^{\alpha }}{\sum_{b=1}^{n}{\left(\sum_{j}a_{ij}\delta_{s_{j}\left(t\right),b}\right)}^{\alpha }}\;.$$

The first term in Equation (3) indicates correct individual sensing and the second term gives the probability of acquiring the correct information through peer communication. Similarly, in Equation (4), the first term corresponds to incorrect sensing that ends up in state *b* and the second term describes acquiring state *b* through peer aggregation. Also note that the system of *N* equations given by (3) and (4) is a mean-field description of the dynamics, which ignores the effect of fluctuations as well as details of possible configurations of beliefs in an agent’s neighbourhood.

The system described above is determined by five parameters: (i) the sensor accuracy *q*, (ii) the complex contagion exponent α for aggregating peer information, (iii) the mix of sensing-aggregating behaviours *p* (or *sensing intensity*), (iv) the volatility of the environment *u*, and (v) the communication structure encoded in the adjacency matrix *A* of a social network between the agents. Note that, while we assume here that all sensors’ accuracy and sensing intensities are equal for all agents, these could also be heterogeneous across the population.

## 3. Results on All-to-All Connected Networks

In this section, we focus only on all-to-all connected networks (or complete graphs) to build an understanding of the basic properties of the above-defined model dynamics via analytical and numerical results. Starting with the simple scenario of a static environment (with u=0), we consider the approximation where all nodes are coupled to the mean-fields $P_{b}\left(t\right)=1/N\sum_{i}P\left(s_{i}\left(t\right)=b\right)$. Subject to this, Equation (3) transforms into

(5) $$\begin{array}{cc} P_{S}\left(t+1\right) & =pq+\left(1-p\right)\frac{P_{S}{\left(t\right)}^{\alpha }}{\sum_{b}P_{b}{\left(t\right)}^{\alpha }}\;,\\ P_{b\neq S}\left(t+1\right) & =p\left(1-q\right)/\left(n-1\right)+\left(1-p\right)\frac{P_{b\neq S}{\left(t\right)}^{\alpha }}{\sum_{b}P_{b}{\left(t\right)}^{\alpha }}\;, \end{array}$$

describing the dynamics of the fractions of correctly and incorrectly sensing agents in a static environment. If we consider the evolution of probabilities at the steady state—and exploiting the symmetry of the problem—the system can be described by a stationary fraction of correctly sensing agents *P* given by

(6) $$P=pq+\left(1-p\right)\frac{P^{\alpha }}{P^{\alpha }+\left(n-1\right){\left[\left(1-P\right)/\left(n-1\right)\right]}^{\alpha }}\;.$$

**Figure 1:**
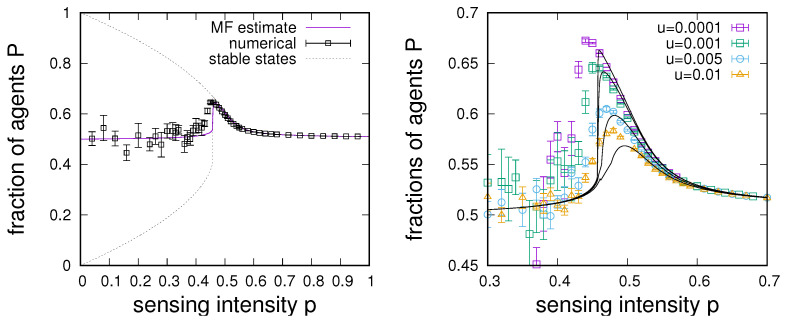
(**Left**) Dependence of the average fraction of correctly sensing agents *P* on the sensing intensity *p*. The figure compares numerical data obtained for an all-to-all connected system with 1000 nodes (black squares) to the mean-field orbit diagram for the bi-stable system and a mean-field estimate for P(p) from Equation (6) (dotted lines) along with an estimate of the stationary outcome of the switching dynamics based on Equation (8) (magenta line). Parameters are as follows: n=2; switching rate u=0.001; α=2; sensing accuracy q=0.51. Numerical data are from simulations with over 10,000 iterations of the dynamical process averaged over 10 independent runs. In the orbit diagram, we find a critical point $p^{*}\approx 0.458$ such that below $p^{*}$, the system is bi-stable and above $p^{*}$, it follows the sensing of all agents and manages to adapt to changing signals. For a switching rate of the external signal of u=0.001, the maximum fraction of agents aware of the correct signal is found at p≈0.466, slightly above the bifurcation point. (**Right**) Comparison of the mean-field estimate (based on Equation (8), black lines) vs. numerical data for different switching rates *u*.

Solutions of Equation (6) transition from a multi-stable phase to a phase with a single attractor. The multi-stable phase is found for sensing intensities *p* smaller than some critical point $p^{*}$ and corresponds to a situation in which most agents lock into a state given by the initial majority state of the collective. In the phase with a single attractor for $p>p^{*}$, agents sense enough on their own such that a majority of the collective will become aware of the correct state of the environment. This situation is illustrated in Figure 1a (in *dotted* lines) for a system with n=2, α=2, and q=0.51, where the critical point is found at around $p^{*}\approx 0.458$ (which was determined numerically).

Operating in either the multi-stable or mono-stable regime has important implications for collective sensing. In the bi-stable phase, if initial conditions are unfavourable, the system may evolve to a state where global sensing is much less accurate than individual sensing (P<q). Moreover, in this phase, the system remains locked in its overall configuration when changes in the environment occur and thus the population is unable to adapt to a dynamical environment.

However, we also note that, when the system is in the $p>p^{*}$ phase, the higher the frequency of direct sensing *p*, the lower the probability *P* of agents to be in the correct state. This occurs since P(p) is monotonically declining with *p* for $p>p^{*}$, and P(p=1)=q. As a consequence, sensing intensities just above the critical point $p^{*}$ result in optimal sensing enhancement from group sensing.

To better explore the behaviour of the system in dynamical environments, we modify the mean-field description to incorporate environmental change. For the sake of tractability, we will approximate the environmental dynamics by a deterministic signal that swaps its state every T=1/u time steps. Subject to these assumptions and using Equations (3) and (4), the average evolution of the system can be approximated by the iteration of Equation (5) and a deterministic version of the change dynamics of the environment (2) in which we deterministically cycle through states in succession

(7) $$S\left(t+1\right)=\left\{\begin{array}{ccc} S(t) & \mathrm{for} & 0<t<T\\ (S(t)\mathrm{mod}n)+1 & \mathrm{if} & t=0 \end{array}\right.,$$

and the average probability *P* of correctly sensing the environment can be found by averaging over the attractor of the recursion relation given above to obtain

(8) $$P\left(t\right)=1/\left(\left(n-1\right)T\right)\sum_{t=T_{0}}^{T_{0}+\left(n-1\right)T}P\left(s\left(t+1\right)=1\right),$$

where $T_{0}$ stands for a transient time and averages are taken over a full cycle through all environments. Mean-field solutions for complete graphs can now be obtained by numerically iterating Equation (5) and then averaging after discarding a transient according to (8).

Results obtained from this mean-field description are shown in Figure 1b, where solid lines give estimates based on Equation (8) for various environmental switching rates *u* and symbols give numerical results obtained from direct simulations of the belief updating dynamics.

As one might have expected, the lower the rate of change *u* of the environment is, the better able the collective is to adapt to the change, and better sensing can thus be achieved for the optimal trade-off points *p*. We also note that this optimal trade-off point shifts to the right with a larger *u*. The above relates to the speed of collectively transitioning to a new state after environmental change, which increases as the intensity of individual sensing increases. Hence, groups with a higher frequency of direct sensing are more robust to changes in the environment at the expense of achieving a weaker enhancement of group sensing, which is a commonly discussed speed–accuracy trade-off in the field of swarm robotics [31].

## 4. Results for Complex Networks

In this section, we explore the dependence of sensing enhancement on the structure of complex networks. As we are particularly interested in exploring the role of degree heterogeneity, clustering, and path lengths, our analysis presented below is focused on four network models. In more detail, we consider (i) random regular graphs (RRG), (ii) Erdös–Rényi random graphs (ER) [32], (iii) Barabási–Albert scale–free networks (BA) [33], and (iv) ring graphs (RG). These choices are motivated as follows. Comparison between results for RRG, ER, and BA will allow us to explore the role of degree heterogeneity. Further, comparing results for RRG and RG—which are both types of regular graphs—will shed light on the implications of spatial embedding.

In the following, we aim to characterize these networks by the amount of sensing enhancement that can be achieved on them. For this purpose, we typically run numerical experiments that directly simulate the stochastic process of sensing and belief change, as detailed in Section 2, and obtain averaged results from between $10^{4}$ and $10^{5}$ updates (depending on *u*). In many of the experiments described below, we are interested in the maximum sensing enhancement achievable on a class of networks. In these, we assume that agents have optimally “tuned” their sensing intensities. Accordingly, we run simulations for a range of sensing intensities to determine P(p) and approximate $P_{max}\left(p_{opt}\right)$, where $P_{max}$ denotes the maximum fraction of the population that can sense correctly, and $p_{opt}$ denotes the sensing intensity at which this value of $P_{max}$ can be realised. We then determine $P_{max}$ and $p_{opt}$ for individual networks and then report the averages of these values over samples from the respective class of network.

As the numerics are relatively costly, experiments presented below are typically evaluated for networks of size N=1000 and—unless otherwise mentioned—averages are calculated over 20 network realisations. When exploring parameter dependencies, we typically focus on a low sensing accuracy close to q=1/(n−1) to investigate sensing enhancement that can be achieved in very adverse settings, when correct sensing only has a small advantage over random selection.

Results presented below also indicate that, depending on the network’s connectivity, optimal sensing can typically be found either on ring graphs or random regular networks. As these topologies can be seen as limiting cases of a Strogatz–Watts-type small world, we also conducted further experiments exploring sensing enhancement as a function of the small-world parameter. To extrapolate between ring graphs and regular random graphs (and thus rule out any effect of degree heterogeneity), we consider a small modification of the small-world model introduced by Watts and Strogatz [34] that allows for the construction of regular small worlds. This is achieved by starting with a ring graph and then picking each link with probability *f* for rewiring. If a link between nodes $x_{1}$ and $x_{2}$ is chosen for rewiring, we also pick another randomly selected link and between nodes $y_{1}$ and $y_{2}$ and swap end nodes, i.e., reconnect $x_{1}$ to $y_{1}$ and $x_{2}$ to $y_{2}$. The procedure introduces random shortcut links exactly as in the traditional small-world model but also ensures that the networks remain regular during the rewiring.

Below, in Section 4.1, we start by exploring sensing enhancement on networks for a binary environment with n=2, ensure the robustness of our findings to different parameter settings, and then proceed by investigating the small-world effect in Section 4.2. The last subsection of the results section generalises findings for n>2 and discusses the dependence on the number of possible states in the environment.

### 4.1. Sensing Enhancement in Binary Environments

**Figure 2:**
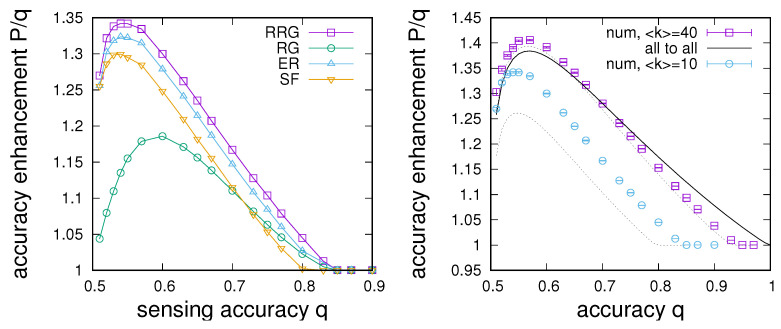
Maximum sensing enhancement P/q as a function of the sensing accuracy *q*. (**Left**): Comparison between numerical data for different types of complex networks for u=0.001, α=2, and 〈k〉=10. (**Right**): Comparison for random regular graphs with connectivity 〈k〉=10 and 〈k〉=40 with the mean-field estimates for limited connectivity (dotted lines) and all-to-all coupling (solid line).

As an initial experiment, we are interested in comparisons of the dependence of sensing enhancement on sensing accuracy between different networks. For this purpose, maximum sensing enhancements $P_{max}\left(q\right)$ have been determined for different sensing accuracies for the four classes of networks discussed above. Results are summarised in Figure 2, where we report maximum sensing enhancements relative to sensing accuracies, i.e., P/q as a function of *q*. More specifically, the left-hand panel of Figure 2 shows the dependence of P/q for networks with 〈k〉=10 for u=0.001 and α=2. We note that the possible sensing enhancement as a function of *q* typically has a maximum for low *q*, allowing for an up to 35% improvement in sensing accuracy depending on the network topology. Additionally, depending on the network structure, sensing enhancement is only possible for a limited range of sensing accuracies, and there typically is a sensing accuracy $q_{c}$ above which sensing enhancement is no longer possible (note the points where P/q=1 in the right-hand panel of Figure 2). Comparing the four different network topologies, we find the largest sensing enhancement for RRGs at around $P_{max}/q\approx 1.35\pm 0.01$, a somewhat lower enhancement for ER with $P_{max}/q\approx 1.33\pm 0.01$ and SF networks with $P_{max}/q\approx 1.3\pm 0.01$, and the lowest enhancement for RGs with $P_{max}/q\approx 1.17\pm 0.01$. The corresponding enhancement cut-offs are $q_{c}\approx 0.83\pm 0.01$ for RRGs, ERs, and RGs and $q_{c}\approx 0.8\pm 0.01$ for SF networks.

Whilst the mean-field description above does not account for sparse connectivities, numerical results (discussed later) show a clear dependence of optimal sensing points on the average degree. To include this effect, we develop a mean-field approximation for finite connectivities that essentially factor in through the exact configurations of states in an agent’s neighbourhood. This effect can be accommodated by modifying Equation (5) via

(9) $$\begin{array}{cc} P_{S}\left(t+1\right) & =pq+\left(1-p\right)\sum_{x_{1},\ldots ,x_{n}}\frac{k!}{\prod_{i=1}^{n}x_{i}!}\prod_{i=1}^{n}P_{i}{\left(t\right)}^{x_{i}}\frac{{\left(x_{S}/k\right)}^{\alpha }}{\sum_{b}{\left(x_{b}/k\right)}^{\alpha }},\\ P_{b\neq S}\left(t+1\right) & =\frac{p(1-q)}{\left(n-1\right)}+\left(1-p\right)\sum_{x_{1},\ldots ,x_{n}}\frac{k!}{\prod_{i=1}^{n}x_{i}!}\prod_{i=1}^{n}P_{i}{\left(t\right)}^{x_{i}}\frac{{\left(x_{b}/k\right)}^{\alpha }}{\sum_{b}{\left(x_{b}/k\right)}^{\alpha }}, \end{array}$$

where sums extend over all $x_{1},\ldots ,x_{n}$, where $x_{i},i=1,\ldots ,n$ represents the number of neighbours holding state *i* at time *t* such that $\sum_{i}x_{i}=k$, and *k* is the connectivity of the network. Evaluating the combinatorics in Expression (9) proves to be computationally too expensive for large *n* and *k*, but can be reasonably accommodated for n=2, where the first expression in (9) transforms to

(10) PS(t+1)=pq+(1−p)∑j=0kkjPS(t)j(1−PS(t))k−j(j/k)α(j/k)α+(1−j/k)α.

Note that the formulations in Equations (9) and (10) assume that all nodes of the graph have roughly equal degrees, and *j* in (10) represents the number of neighbours in state s(t)=S(t). Equation (10) can then be used to adapt Equation (5) for limited degrees and can be inserted into Equation (8) to yield estimates for the fraction of agents in the possession of correct information. Estimates based on Equation (10) are compared to numerical data in the right-hand panel of Figure 2, where we see that, whereas there are considerable differences between mean-field estimates and numerical data for small connectivity, reasonable agreement is found for connectivities above around 〈k〉=40, and the main trends of the dependencies in the numerical data are captured by the limited connectivity mean-field approach (dotted lines). This contrasts with the all-to-all-coupling-based mean-field approach (solid line), which fails to account for the observed limited range of sensing accuracies up to $q_{c}$ for which consensus enhancement is possible.

We thus see that the structure of the social network connecting a population can have a strong impact on achievable sensing enhancement. Results presented in Figure 2 (left) suggest that, in otherwise random networks, heterogeneity plays a major role, seemingly impeding sensing enhancement. However, comparison between results for RRGs and RGs also shows strong differences, which suggests that degree regularity on its own also does not ensure the best performance.

**Figure 3:**
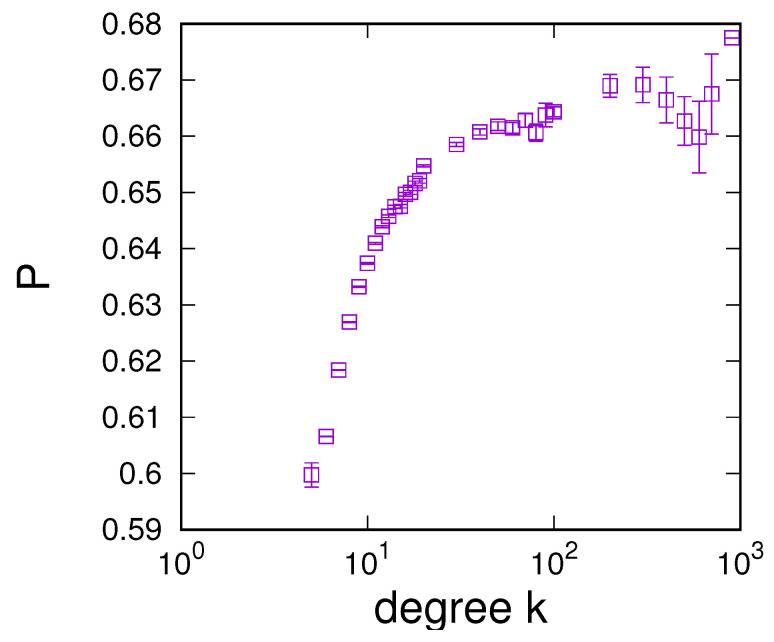
Average probability to sense the correct state of the environment *P* as a function of node degree *k* averaged over 10 Barabasi–Albert-type SF networks with $N=10^{4}$ for u=0.001, α=2, q=0.51, p=0.31, and 〈k〉=10. Error bars give standard errors.

To further explore the role of degree heterogeneity, it proves instructive to investigate the dependence of the probability to accurately sense the environment on node degree. We have evaluated this relationship for SF networks, and results are plotted in Figure 3. As one would expect, we see that hub nodes tend to have better awareness of the environment than low-degree nodes. However, after an initial steep increase, increases tend to quickly saturate with degree from k≈20 on. We thus see a reason for the poorer performance of the SF networks compared to RRGs: connectivity spent on hub nodes only yields a small improvement in sensing, which comes at the cost of a relatively larger deterioration of the availability of correct information at low-degree nodes.

**Figure 4:**
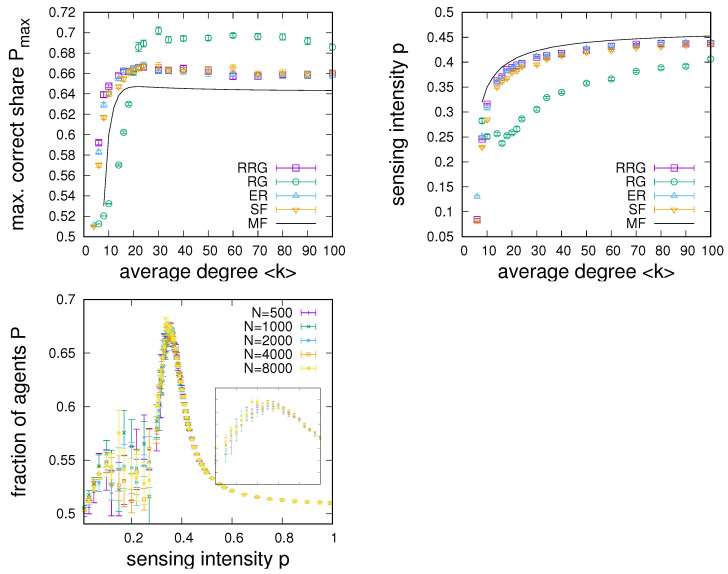
Dependence of the point of optimal sensing enhancement on connectivity for different networks. (**Left**): Optimal sensing $P_{max}$ vs. connectivity 〈k〉. (**Right**): Required sensing intensity at the optimum vs. connectivity. (**Bottom**): Analysis of the P(p) dependence for RGs for different system sizes ranging from N=500 to N=8000, where the inset magnifies the region 0.4≤p≤0.5. Numerical data obtained from simulations of $10^{4}$ iterations of the updating process and averaged over 20 networks of size N=1000 (for the first two panels) for q=0.51, α=2, and u=0.001. The black lines give mean-field estimates.

The observation of the degree dependence of sensing in Figure 3 motivates a more detailed investigation of sensing enhancement on network connectivity. Figure 4 compares the dependence of optimal sensing on the connectivity of networks and mean-field estimates based on Equation (10), both in terms of the optimal sensing enhancement in Figure 4 (left) and for the sensing intensity *p* required at the optimal point in the panel of Figure 4 (right). These results lead to a number of observations. First, close inspection shows that larger connectivity does not always lead to improved sensing. In contrast, there exists a connectivity at which sensing is maximally enhanced at around 〈k〉=20 for RRGs, ERs, and SF networks and at roughly 〈k〉=30 for RGs. The existence of such a connectivity maximum is also reproduced in the mean-field estimate, which fits reasonably well with results for RRGs, ERs, and SF networks, for which differences become very small for large connectivities. Nevertheless, for low connectivity 〈k〉<20, we note that generally RRGs perform better than ERs, which are in turn superior to SF networks and RGs, which perform significantly poorer in terms of sensing enhancement. Differences between these networks tend to become larger as the connectivity of the graph decreases. For 〈k〉>20, the order of performance is maintained for RRGs, ERs, and SF networks, but it is apparent that, for large connectivities, RGs have a clear advantage. This advantage manifests itself in terms of a clearly superior performance, but also in terms of much reduced sensing intensities required at the optimal point—see Figure 4 (left). For the setting investigated in Figure 4, we also note that sensing enhancement is only possible for 〈k〉≥6; otherwise, no sensing enhancement is found.

In particular, noticing the connectivity dependence of the performance of ring graphs, one might be inclined to speculate that improvements in sensing for larger connectivity could result from the corresponding reduction in the average shortest path length. In this case, if sensing enhancement was strongly influenced by the average shortest path length, one would also expect drastic changes when the system size of ring graphs is varied. However, analysis of the P(p) dependence for RGs of varying sizes in Figure 4 (bottom) shows no significant dependence on system size over a range of system sizes in which the average shortest path lengths of ring graphs vary by a factor of at least 10. This analysis is further backed up by a more detailed analysis of the dependence of optimal enhancement on the system size in Appendix A.1. There, we clearly see that the point and amount of optimal enhancement become largely independent of system size for $N\geq 10^{3}$, showing that average shortest path lengths are not a strong determinant of a network’s sensing enhancement. Results for the other network types show a similar independence of system size for a large *N* (see Appendix A.1 for details).

**Figure 5:**
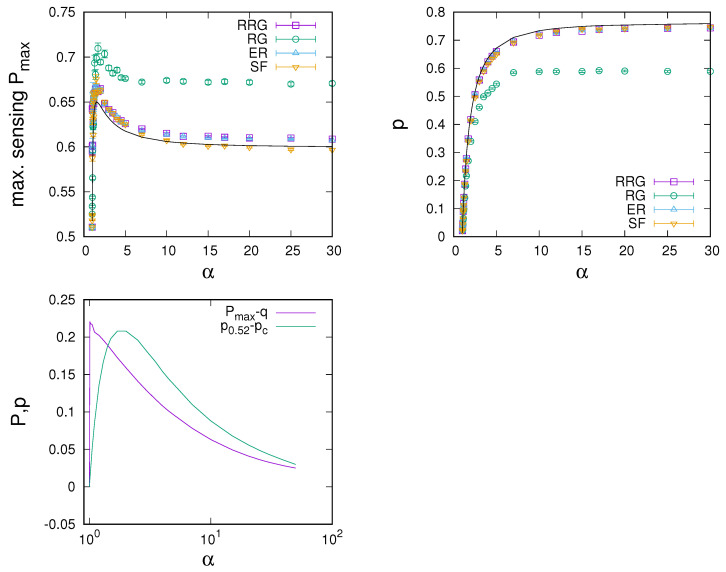
Dependence of the point of maximum sensing enhancement on α. (**Left**): Dependence of optimal sensing $P_{\max}$ on α for various networks with 〈k〉=40, u=0.001, and q=0.51. There is a max value of α, such that sensing is maximally enhanced. (**Right**): Dependence of the required sensing intensity *p* at the maximum point on α for various complex networks. The solid line gives the mean-field estimate. (**Bottom**): Results from the mean-field analysis for the dependence of $P_{\max}$ and the width of the enhancement region $p_{P>0.52}$ on α. More consensus enhancement is possible for a smaller α>1, but the width of the peak converges to zero and transients become longer as one approaches α=1 from above.

As a last parameter dependency of interest, Figure 5 shows numerical results for the effect of the selection strength α for networks with 〈k〉=40 (where 〈k〉=40 has been chosen, as, for this connectivity, the sensing enhancement for all network types has been roughly saturated with degree). As for the dependence on connectivity—see Figure 5 (left)—we again note the existence of an optimal α at which the largest sensing enhancement is possible. For RRGs, ERs, and SF networks, optimal α is found at around α≈1.5, which roughly coincides with the mean-field prediction; for RGs, the optimal α is located at a slightly larger α≈1.7. From the α dependencies, we also see that the order of network performance is consistently maintained, i.e., for 〈k〉=40, RGs allow for more enhancement than RRGs, ERs, and SF networks, differences between which become small. As a last observation from Figure 5 (left), we also see that sensing enhancement is only possible for α>1, i.e., only in the presence of complex contagion and not in linear voter-like dynamics with α=1 [35].

Figure 5 (right) continues the analysis by showing the dependence of the required sensing intensity at the optimal point on α. We again note that differences between RRGs, ERs, and SF networks are rather small and close to the mean-field expectation. In contrast, and in particular for larger α, sensing intensities required at RGs are noticeably smaller.

Further, results from an analysis of the finite-connectivity mean-field orbit diagram are presented in the bottom panel of Figure 5. In more detail, we plot the sensing enhancement at the bifurcation point as a function of α. One notes that the closer α is to α=1 from above, the larger the possible sensing enhancement, which would suggest an optimum at the smallest possible α approaching α=1. However, the smaller the α, the slower the response dynamics after a switch in the environmental signal. Hence, as argued earlier, points of optimal sensing will generally be found at slightly larger values than the sensing intensity at the bifurcation point. Values will be larger with a shorter time scale of environmental change. Figure 5 (bottom) also shows that the size of the parameter region of sensing intensities for which meaningful sensing enhancement is possible quickly converges to zero as α approaches one. This in effect limits the possible sensing enhancement for small α and explains the existence of an optimal value of α distinctly larger than α=1—again an effect related to speed–accuracy trade-offs [31].

### 4.2. Small-World Effects

Results in Section 4.1 have shown that maximum sensing enhancement can be achieved on either random regular graphs for low connectivity or ring graphs for large connectivity. Both types of networks can be seen as limiting cases of regular small-world networks, which we investigate in more detail in this subsection. For this purpose, regular small worlds with varying shortcut density *f* have been constructed.

**Figure 6:**
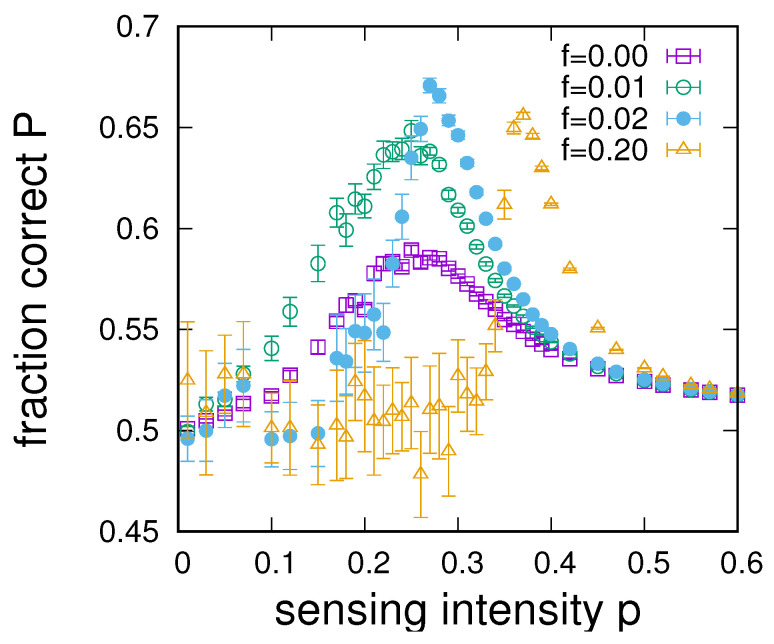
Dependence of the average fraction of correctly sensing agents on the sensing intensity from simulations on small-world networks with different small-world parameters f=0,0.01,0.02 and f=0.2 for n=2, q=0.51, α=2, u=0.001, and 〈k〉=16. Data points provide averages over 20 small-world networks of size N=1000.

Figure 6 illustrates typical results for the dependence of the fraction of the populations that senses correctly *P* on the sensing intensity *p* for different settings of the small-world parameter *f*. These numerical results highlight that optimal sensing is not achieved in either of the limiting cases of f=0 (an RG) or f=1 (an RRG), but instead at some intermediate value of *f*. The settings for *f* chosen in Figure 6 indicate the existence of an optimal small world for sensing enhancement.

**Figure 7:**
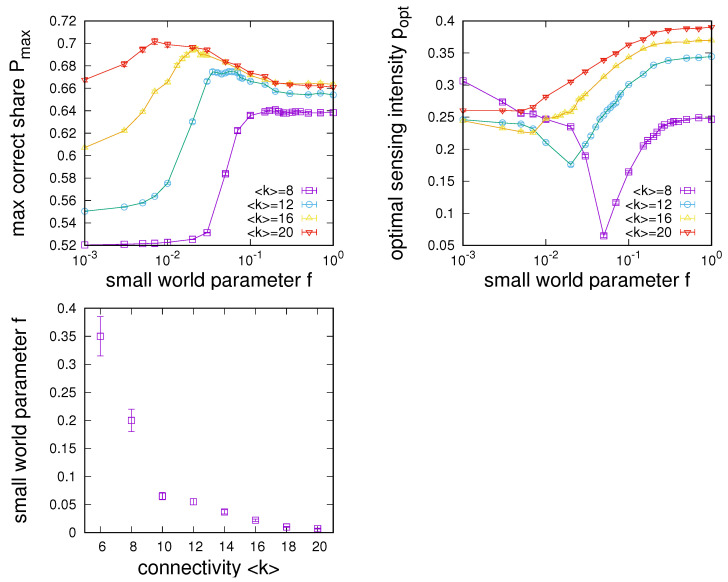
Dependence of the maximum achievable share of correctly sensing agents $P_{max}$ (**left**) and the sensing intensity at which this maximum is achieved (**right**) on the small-world parameter *f* used to construct the social network for networks of different average connectivity 〈k〉=8 to 〈k〉=20. The (**bottom**) left panel yields the dependence of the optimal small-world parameter *f* for which sensing enhancement is maximised on the average degree. Data obtained from simulations on regular small-world networks of size N=1000 for n=2, α=2, u=0.001 and q=0.51. For each data point, 50 small-world networks were constructed, and for each, the optimal sensing intensity and maximum of P(p) were determined and then averaged. We see that optimal sensing enhancement can be achieved for networks in the small-world region (for small *f*).

This idea is further explored in the experiments showcased in the panels of Figure 7. Here, we constructed small-world networks for different settings of the small-world parameter *f* and then determined the optimal sensing intensity $p_{opt}$ and the maximum fraction of correctly sensing agents $P_{max}=P\left(p_{opt}\right)$ for each network. We plot the dependence of $P_{max}$ (left-hand panel) and $p_{opt}$ (right-hand panel) on *f*, again for different choices of the network connectivity 〈k〉 to ensure robustness of our findings. We make several observations from these results.

First, as expected, small worlds are found to interpolate between the settings of an RG and an RRG. As RGs provide hardly any sensing enhancement for low connectivity in the regime where RRGs performed best (as observed in Section 4.1), we see a corresponding transition between both networks for low connectivity. As, e.g., seen for 〈k〉=8 or 〈k〉=10, we find that a relatively small density of shortcuts is typically sufficient to transform an RG to achieve RRG-like performance. In this transition region towards RRG-like performance, a minimum of required sensing intensities is assumed (see Figure 7, top right).

Second, the interpolation between the RG and the RRG regimes is not just achieved through a monotonic increase or decrease. Instead, we see a clear maximum in sensing enhancement for small *f*, indicating that there is a low shortcut density in the small-world region is optimal for sensing enhancement. The latter point is further supported by the bottom left panel of Figure 7, which shows the dependence of the small-world parameter that maximises sensing enhancement on the average degree. The results show that this typically happens for small densities of shortcuts *f*, which decrease as the network’s connectivity increases.

**Figure A2:**
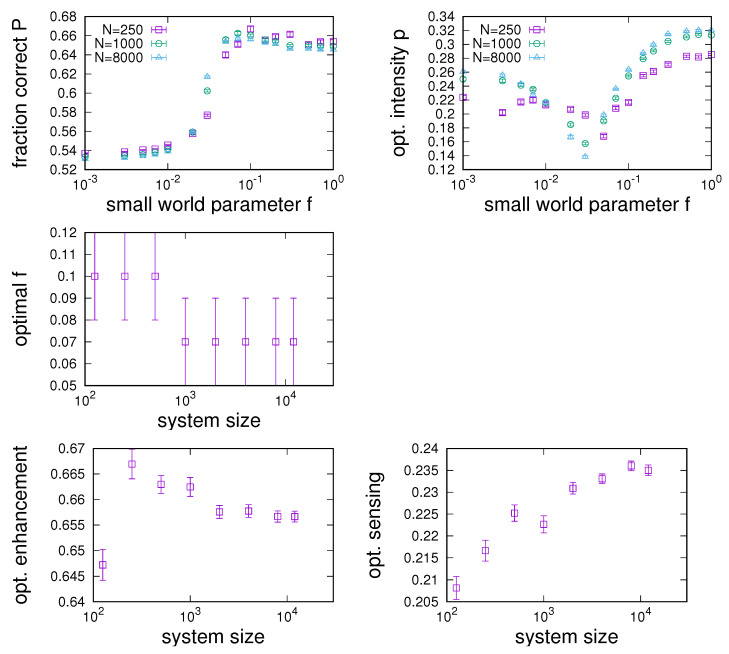
(**Top**) Dependence of the optimal sensing enhancement $P_{\max}$ (**left**) and optimal sensing intensity (**right**) on the small-world parameter *f* for different system sizes N=250, N=1000, and N=8000. (**Middle**) Dependence of the optimal small-world parameter on system size (error bars given by discretisation of *f*). (**Bottom**) Dependence of the optimal enhancement at the optimal small-world parameter (**left**) and sensing intensity at which this can be realised (**right**) on system size. Data points are for q=0.51, u=0.001, α=2, and 〈k〉=10, and points in the figures represent averages over 50 network configurations.

We have also carried out experiments to investigate the influence of system size on the location of the optimal small-world region. To not disrupt the flow of the narrative, more detailed results are presented in Appendix A.2. Results presented in Figure A2 indicate that the optimal small-world parameter *f* is roughly independent of system size for sufficiently large small worlds of size $N\geq 10^{3}$.

**Figure 8:**
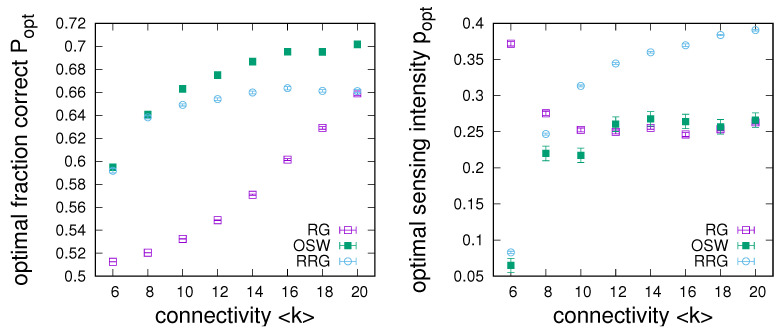
Comparison between the dependencies of maximum achievable sensing enhancement (**left**) and the required sensing intensity at the optimum (**right**) on network connectivity between ring graphs (RGs), regular random graphs (RRGs), and optimal small worlds (OSWs). Data obtained from averages over 50 simulations on networks of size N=1000 for n=2, α=2, u=0.001, and q=0.51.

As a last point in this subsection, Figure 8 compares the achievable sensing enhancement on optimal small worlds to ring graphs and regular random graphs. On the one hand, in the left-hand panel of Figure 8, we see that optimal small worlds (OSWs) only have a small advantage over the limiting cases of RRGs and RGs for small connectivity, but can lead to substantial improvements for larger connectivity. On the other hand, the right-hand panel of Figure 8 underlines that this improved performance can generally be achieved by a sensing intensity, which is at worst only marginally larger than the minimum required for either RRGs or RGs.

### 4.3. Effects of Non-Binary Environments

In the above, we have investigated the role of networks for sensing enhancement in binary settings. Here, we generalize from the scenario of two possible states of the environment to the general case of an arbitrary number of states n≥2.

**Figure 9:**
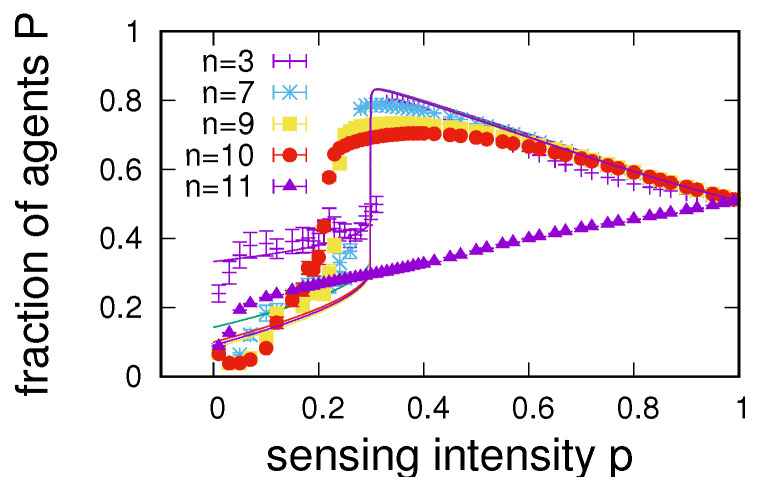
Dependence of the fraction of correct agents *P* on the sensing intensity *p* in environments with different numbers of states *n*. Results are from numerical simulations for RRG networks of size N=1000 averaged over 20 networks (symbols) and from mean-field estimates (solid lines). Other parameters are q=0.51, u=0.001, α=2, and 〈k〉=60.

First results are presented in Figure 9, in which we compare simulation results obtained for different *n* to mean-field estimates based on Equation (5). In principle, numerical results are found to be similar in behaviour to our observations in Figure 1a, again typically showing a low sensing region in which correct sensing is roughly given by P≈1/n, separated by a bifurcation of the multi-stable system from an enhancement region in which P>q. However, we also note a clear dependence of the region of maximum enhancement on the number of environmental states, where increasing *n* typically results in a decline in enhancement until no enhancement is possible for an *n* larger than a certain maximum number of states $n_{\max}$. In Figure 9 for q=0.51 on an RRG, we find $n_{\max}=11$, i.e., for this particular value of *q*, no enhancement is possible in an environment allowing for more than 10 states. We also observe that, with a growing number of states, the region of maximum enhancement tends to move towards lower sensing intensities (which is abruptly reversed for $n\geq n_{\max}$, for which the system no longer exhibits a bifurcation). One further notes that, for n>2, although capturing qualitative behaviours, the mean-field description fails to capture the shift in bifurcation points.

**Figure 10:**
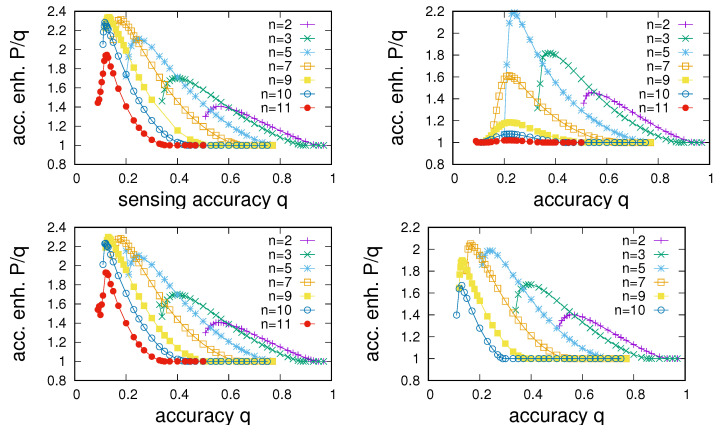
Comparison of the dependence of the largest accuracy enhancement P/q on the sensing accuracy *q* for different numbers of environmental states *n*. From left to right and top to bottom, panels give data for RRGs, RGs, ER-type random networks, and scale-free networks (note that n=11 was not plotted for SF networks, because no enhancement is possible in that case). Data averaged from 20 realisations of networks of size N=1000. Other parameters are u=0.001, α=2, and 〈k〉=40.

**Figure 11:**
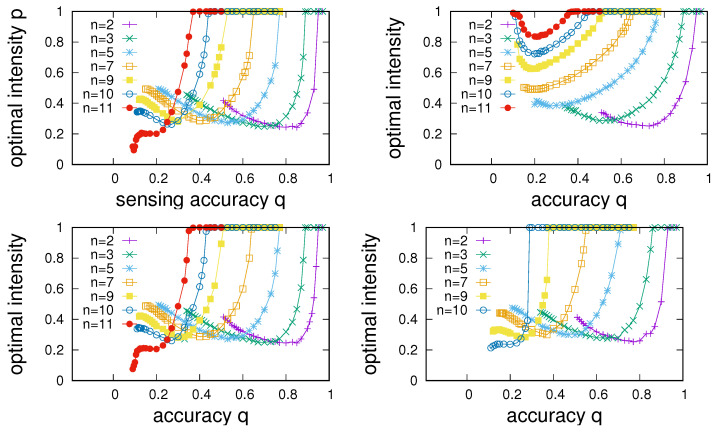
Comparison of the dependence of the sensing intensity for which the largest accuracy enhancement can be achieved (see Figure 10) on the sensing accuracy *q* for different numbers of environmental states *n*. From left to right and top to bottom, panels give data for RRGs, RGs, ER-type random networks, and scale-free networks. Parameter settings as in Figure 10.

To proceed, we aim to quantify differences in the network structure in multi-state environments. For this purpose, we have run numerical experiments in which we have determined the P(p) dependence and extracted points of maximum sensing enhancement P/q for each setting of *q* for the four different types of networks discussed in Section 4.1 and different settings of *n*. Results of these experiments are summarised in Figure 10, where the dependence of the points of maximum sensing enhancement on *q* is shown for the four different networks. The corresponding information about sensing intensities at which maximum enhancement is achieved is given in Figure 11. A number of observations are in order.

First, we note that sensing enhancement typically depends on *q* and tends to reach a maximum as a function of *q* and then decline to P/q=1 in a region in which sensing enhancement becomes impossible. Determining the largest value of $q_{max}$ at which P/q>1 allows us to characterize the extent of the region of sensing accuracies for which sensing enhancement can take place.

As a second point, we note that the point of maximum enhancement also is a function of the number of states. Maximum enhancement is observed to first increase with *n*, reaches a maximum as a function of *n*, and then declines. In agreement to what we noted in Figure 9, we see that enhancement becomes impossible beyond a certain number of environmental states. The experiments presented in Figure 10 allow us to determine an overall $n_{max}$ for each of the four networks; we find $n_{max}=12$ for ER networks and RRGs and find $n_{max}=11$ for SF networks and RGs.

Third, comparison between the panels in Figure 10 shows clear differences among network types. Whereas, as one might expect, dependencies for ER and RRGs are very similar, SF networks and RGs show distinctly different behaviour. Two points stand out. First, SF networks are found to always show slightly poorer enhancement compared to ERs and RRGs. Second, whereas maximum enhancement for RRGs, ERs, and SF networks is found for values of *n* just below $n_{max}$, the best enhancement for RGs tends to occur for substantially smaller *n*.

**Figure 12:**
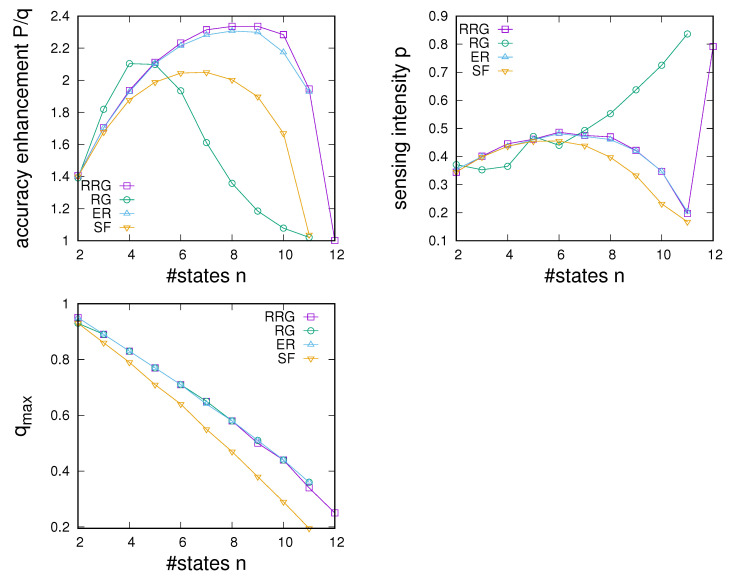
Comparison of the dependence of maximum achievable accuracy enhancement (**top left**), the sensing intensity at which the optimal accuracy enhancement can be achieved (**top right**), and maximum sensing accuracy for which sensing enhancement can be achieved (**bottom**) on the number of environmental states for RRGs, RGs, ER-type networks, and scale-free networks. Dependence of Parameter settings as in Figure 10 and Figure 11.

To emphasise the above points, we have characterised each network type by its dependence on (i) the maximum achievable accuracy enhancement as a function of *n*, the sensing intensity required to achieve this enhancement as a function of *n*, and the maximum extent $q_{max}$ of the region of sensing accuracies for which enhancement is possible as a function of *n*. Results are given in Figure 12. We again see different propensities of networks to enhance sensing. Note that RGs tend to allow for better enhancement for a low number of states, whereas RRGs and ER-type networks give better results for larger numbers of states (see Figure 12 right). This is also reflected in much lower sensing intensities required to achieve optimal results for the latter networks (see Figure 12 right). SF networks, on the other hand, always exhibit poorer performance and also tend to allow for a smaller range of sensing accuracies *q* for which enhancement is possible (see Figure 12 bottom).

## 5. Summary and Conclusions

In this paper, we have investigated the role of the structure of social network topologies on a collective’s capacity for amplifying the accuracy of individual sensing through aggregating peer information. Previous work has explored the problem in unstructured or spatially embedded populations and has shed light on the nature of the underlying mechanisms and the required trade-offs between noise in communication, individual sensing, and information aggregation from peers [21,22]. Differently, our main focus here has been on the dependence of enhancement and optimal parameters on network structure. Our results clearly show that different structures of social communication networks influence a collective’s potential for sensing enhancement.

As a first point, we have shown that network heterogeneity in the form of degree heterogeneity generally impedes a collective’s average sensing enhancement. Whereas the presence of very strongly connected individuals in the form of hub nodes provides an information advantage to these individuals, their advantage tends to come at the cost of poorer information availability to agents at the periphery of the network. As a consequence, when comparing networks of the same average degree, the marginal benefit of adding connections to hub nodes does not outweigh the losses incurred on lower connectivity nodes. This effect strongly resembles similar observations in other consensus-finding problems, as, e.g., studies on optimal synchronisation [36,37].

Second, our findings have also highlighted the role of connectivity. Whereas we find that—again, similar to results established in the context of optimal synchronisation [37]—regular random networks provide very strong potential for sensing enhancement for low connectivity, we also find that—unlike in synchronisation problems, where spatial structure tends to impede synchronisation [34]—ring graphs tend to have much better enhancement potential than random networks for large connectivity. Interestingly, since this observed behaviour on ring graphs seems independent of system size, this effect does not seem to be a function of a reducing average shortest path length with increasing connectivity.

Third, we have investigated sensing enhancement on Watts–Strogatz-type small-world networks and have shown that optimal sensing enhancement can typically be found in the so-called small-world region, i.e., for a relatively small density of shortcut links. Our finding in this regard is closely connected to the trade-off between the requirements for separation and integration discussed relating to the organisation of brain networks [38,39]. Similar to the neuroscience context, one could speculate that also small-world networks in our context realize an optimal trade-off between the separation and independent processing of information in local communities and the integration of independent views from the local level to benefit the collective as a whole.

As a fourth point, we have also investigated the effects of multi-state environments on sensing enhancement on complex networks. Results here have again pointed out the detriments of degree heterogeneity but have also shown that, up to a threshold number of states, random graphs tend to perform relatively well when there are more states in the environment, whereas ring graphs tend to perform poorly in environments with many states.

Beyond exploring network characteristics, our findings have also pointed towards the important role of the selection strength exponent α, indicating that optimal enhancement can typically be achieved in the region of 1<α<2. This finding might indicate that the majority rule that is typically applied in the robotics context [19] might not necessarily be the best choice.

Our paper is also subject to a number of limitations. First, even though we have presented a framework that is strongly simplified in comparison to the work of [21,22], the resulting model is still characterised by a number of parameters. Even though we have systematically explored the influence of the main parameters, computational complexities have prohibited a full factorial design. As a second point, our study has been restricted to undirected unweighted networks. It seems an interesting avenue for future research to explore more general settings in this regard, where one might be particularly interested in the effects of hierarchical organisation on sensing enhancement [39].

Last but not least, we also note that the model developed in the context of sensing and opinion sharing in the present paper has similarities to models developed in the context of influence maximisation for voter dynamics [40,41], where individual sensing could be perceived as a connection to an external zealot [42], whose state represents the environment. One might wonder if continuous optimisation techniques as used in [43] could then be used to explore optimised individual sensing on complex networks.

## Figures and Tables

**Figure A1:**
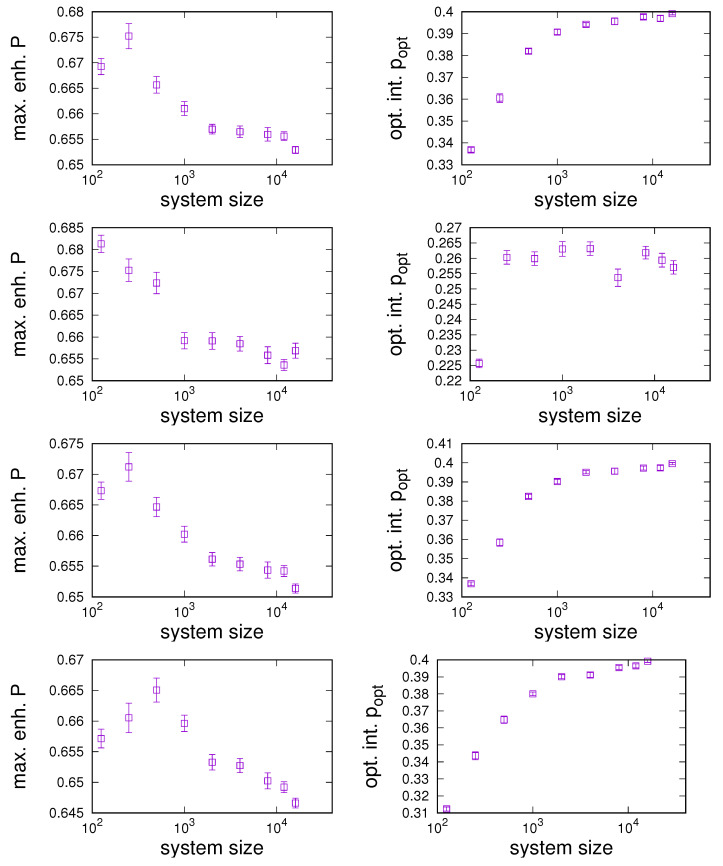
Dependence of the optimal achievable sensing enhancement $P_{max}$ (**left**) and the sensing intensities at which it can be realised (**right**) on the system size for different types of networks (RRG, RG, ER, and SF from top to bottom). Data points are for q=0.51, u=0.001, α=2, and 〈k〉=20, and points in the figures represent averages over 50 network configurations.

## Data Availability

Not applicable.

## Appendix A. Dependence on System Size

As supplemental information, we provide a more detailed analysis of the scaling with system size. In Appendix A.1, analysis of the dependence of optimal enhancement and optimal sensing intensities for different network types is presented. Appendix A.2 presents more results analysing the dependence of the optimal small-world parameter on system size.

### Appendix A.1. System-Size Dependence of Optimal Enhancement and Intensity

In this subsection, we provide a more detailed analysis of the scaling of the optimal enhancement $P_{\max}$ and the optimal sensing intensity $p_{\mathrm{opt}}$ with system size. Data have again been obtained from numerical simulations in which the stochastic dynamics has been simulated for a range of sensing intensities *p* to determine optimal values, which have then been averaged over 50 independent network realisations. Results of this analysis are presented in Figure A1, where the dependence of the maximum enhancement on system size is given in the left-hand panels and the dependence of optimal intensities in the right-hand side panels.

Careful analysis of the data in Figure A1 shows some dependence of optimal points on the system size, in particular for very small system sizes of N≤500. Shifts in the location of optimal intensities are found to be more pronounced than shifts in optimal enhancement. However, for all network types investigated, we note clear indications of saturation of quantities with system size and no strong further shifts of quantities for system sizes of N≥1000. In particular, for the case of ring graphs (second row in Figure A1, we note no statistically significant dependence on a system size beyond N=1000).

As a consequence, for computational reasons, all experiments presented in the main text have been run for N=1000, beyond which strong finite size effects can be ruled out.

### Appendix A.2. System-Size Dependence of the Optimal Small-World Parameter

In this subsection, we provide additional results showing the dependence of the optimal small-world parameter on the system size with results shown in Figure A2. Investigating the dependence of optimal sensing enhancement (Figure A2 (left)) and optimal intensities (Figure A2 (right)) for different system sizes, we note the presence of some finite-size effect for small system sizes, but also note that dependencies become largely independent of system size for sizes of N≥1000. This observation is supported by numerical results showing the dependence of the optimal small-world parameter on the system size in the middle panel of Figure A2. Moreover, the optimal enhancement at the optimal small-world parameter (Figure A2 (bottom, left)) becomes largely independent of system size beyond N=1000. In contrast, optimal intensities in the optimal small-world regime show a marginal dependence on system size (cf. Figure A2 (bottom, right)).
